# Therapeutic Plasma Exchange in Postural Tachycardia Syndrome (POTS)

**DOI:** 10.7759/cureus.91804

**Published:** 2025-09-07

**Authors:** Yigit Baykara, Yamac Akgun

**Affiliations:** 1 Department of Pathology, University of Arizona, Tucson, USA; 2 Department of Pathology and Laboratory Medicine, University of Miami Miller School of Medicine, Miami, USA

**Keywords:** autoantibodies, autonomic neuropathy, immune dysfunction, plasma exchange, postural tachycardia syndrome

## Abstract

Postural tachycardia syndrome (POTS) is a complex autonomic disorder with growing evidence suggesting an autoimmune contribution in a subset of patients. Therapeutic plasma exchange (TPE) is known as a potential treatment option, particularly for patients with severe or refractory disease. This editorial summarizes the clinical features of POTS, the pathophysiologic rationale for plasma exchange, and the current evidence supporting its use. Although encouraging, there is a need for larger studies and standardized protocols. Further research is warranted to clarify patient selection, treatment regimens, and long-term outcomes.

## Editorial

Postural tachycardia syndrome (POTS) is an autonomic nervous system condition that is characterized by an increase in heart rate of at least 30 beats per minute (40 beats per minute in adolescents or children) upon standing from the supine position without corresponding hypotension [1]. POTS is associated with symptoms of orthostatic intolerance such as dizziness, lightheadedness, palpitations, fatigue, headache, difficulty concentrating, and blurred vision. However, additional symptoms such as gastrointestinal symptoms, myalgias, dyspnea, and chest pain can also be seen [2]. POTS primarily affects women of reproductive age [3]. It is believed that POTS affects between 500,000 and 3,000,000 people in the United States [4]; however, its true prevalence remains unknown to date. Unfortunately, there are no standardized diagnostic tests or treatment options for patients with POTS, and the main goal of pharmacologic therapy has been symptomatic relief of orthostatic intolerance with drugs such as blood volume expanders, heart rate inhibitors, vasoconstrictors, and sympatholytics [3]. Although there is insufficient evidence, due to the possible autoimmune mechanisms involved in POTS generation, several case reports and case series have supported the potential benefit of intravenous immunoglobulin (IVIg) and/or plasma exchange in the treatment of POTS [5-9], with an ongoing clinical trial for the former.

Pathophysiology of postural tachycardia syndrome

Although the pathophysiology of POTS remains complex and not fully understood, several studies suggest the possibility of an immunological cause in a significant portion of the cases [1,3,5,6,10], which might be triggered by stressors such as viral infections, trauma, pregnancy, or surgery [3]. Immune dysfunction caused by the aforementioned factors might lead to the formation of autoantibodies against adrenergic, cholinergic, and acetylcholine receptors [5], henceforth paving the way to autoimmunity. Both specific and non-specific antibodies have been identified in patients with POTS, including autoantibodies directed against G-protein coupled membrane receptors, which can give rise to the symptoms [6], as the receptor is a critical component of the autonomic nervous system and modulates the function of adrenergic and muscarinic receptors [10]. Autoantibodies might be detected in some patients, and around one-fifth of the patients report a history of an autoimmune disease at presentation [1,3].

Mechanism of action of therapeutic plasma exchange

**Figure 1:**
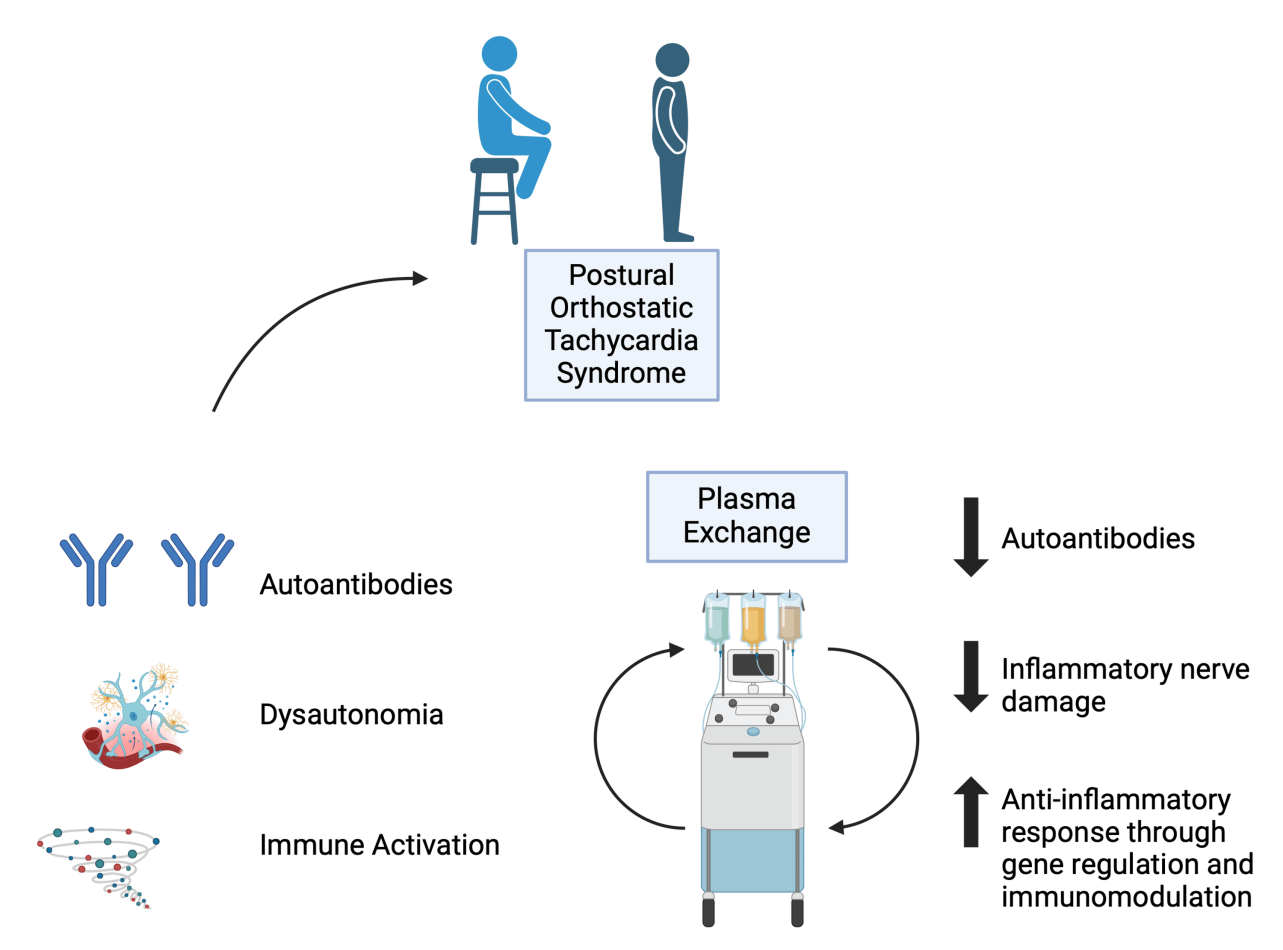
Pathophysiology of postural tachycardia syndrome and the mechanism of plasma exchange Image Credit: Yamac Akgun; Created with BioRender.com.

Therapeutic plasma exchange (TPE) is a procedure that removes circulating antibodies by exchanging the patient’s plasma with fresh plasma or albumin solution. Since in a significant portion of cases, there are specific and non-specific antibodies detected, TPE can be of help by removing the circulating autoantibodies in the body in POTS of autoimmune origin (Figure 1). Decreased concentration of autoantibodies underlying the disease process might help alleviate the symptoms of the disease.

Evidence supporting therapeutic plasma exchange in POTS

There are many reports supporting the benefit of TPE in the treatment of POTS.

Kesterson et al. reported in their series that two patients with severe and refractory POTS experienced significant clinical and functional improvement after TPE, even though the antibody test was negative for both patients [6]. One of those two patients reported further improvement with the addition of IVIg. Blitshteyn and Brook described a patient who developed POTS with anti-NMDA receptor antibodies following human papillomavirus vaccination and whose symptoms improved significantly after TPE and prednisone treatment [7]. Following the treatment, a repeat anti-NMDA receptor antibody test was done, which was negative.

Wells et al. presented a patient with POTS following an episode of shingles whose symptoms improved after a trial of TPE; however, her symptoms returned four weeks after the cessation of TPE, and she was put on maintenance therapy with TPE with a positive response [5]. Clark and Davis reported a pediatric patient with pandysautonomia and POTS who promptly responded to TPE [8]. When their patient's symptoms were less severe, she was switched to IVIg alone, to which she responded poorly; repeat TPE was initiated, and the patient was put on monthly prophylactic TPE. Their patient was negative for known autoantibodies at the initial presentation; however, about two years after the initial presentation (a year after the initiation of repeat TPE), a repeat antibody test revealed anti-ganglionic neuronal acetylcholine receptor antibody. Similarly, Seeley et al. reported the case of a patient with long-COVID-related POTS and autoimmune neurological dysfunction who achieved neurologic improvement following a course of TPE treatment [9].

The positive responses of patients to the treatment with TPE indicate the presence of immune dysfunction and the role of autoantibodies as the culprit in both antibody-proven and antibody-negative cases. Furthermore, even though the initial antibody screen might be negative, repeat testing may reveal the autoantibodies involved during the course of the disease.

Challenges and future directions

The use of TPE seems to show promising results, with improved symptoms of POTS; however, there are several challenges and limitations to overcome. Firstly, despite its prevalence and significant impact on quality of life, POTS remains underdiagnosed and misunderstood, posing challenges for both patients and healthcare providers because of the lack of biomarkers that can show the presence, activity, and severity of the disease [10]. Moreover, as POTS has a complex and intricate pathophysiology, not all patients with POTS may have an underlying autoimmune process with autoantibodies. Even more complicated, autoantibodies may not be detected at the time of presentation, even though they are what is causing the symptoms, or they may present in the circulation without contributing to the disease process. Furthermore, some patients who appear to respond to TPE might have improved spontaneously in temporal association with the intervention or as a result of a placebo effect. Future studies are required to establish its definitive efficacy and to identify clinical biomarkers that can aid in selecting patients for TPE treatment in autoimmune-mediated POTS. In addition, there is no standardized TPE protocol for patients with POTS regarding the frequency, duration, and replacement fluid used, which precludes the implementation of TPE with maximum efficacy for the patients. Finally, the cost-effectiveness of TPE in the treatment of POTS should be thoroughly evaluated, and its potential benefits must be weighed against possible complications such as infection, bleeding, hypotension, vascular access issues, and hypocalcemia. 

TPE might have a potential utility in treating POTS either as a standalone therapy or in combination with other immunomodulatory drugs/symptomatic treatment. As TPE removes the antibodies in circulation, it may alleviate the symptoms of POTS of autoimmune origin. It may be especially beneficial in patients with severe and refractory POTS. However, there are still lots of unknowns regarding treating POTS patients with TPE due to the lack of ample evidence and randomized clinical trials. Further studies are warranted to explore the benefits and risks of this potential treatment modality, to delineate the indications, and to standardize the treatment. Establishing guidelines for the use of TPE for POTS is especially at utmost important for patients with refractory disease. This new treatment modality might improve their quality of life and reduce the morbidity related to POTS.
